# Improving Folic Acid Supplementation Through Electronic Medical Record Interface Modifications—A Retrospective Study

**DOI:** 10.3390/jcm14144939

**Published:** 2025-07-11

**Authors:** Dina Litvak, Eugene Merzon, Yotam Shenhar, Ilan Green, Shlomo Vinker, Ariel Israel, Avivit Golan Cohen

**Affiliations:** 1 Department of Family Medicine, Faculty of Medicine, Tel Aviv University, Tel Aviv 6910127, Israel; dinalitvak@mail.tau.ac.il (D.L.); igreen@leumit.co.il (I.G.); svinker@leumit.co.il (S.V.); aisrael@leumit.co.il (A.I.); 2 Leumit Health Services, 23 Shprinzak St., Tel Aviv 6473817, Israel; emarzon@leumit.co.il (E.M.); yshenhar@leumit.co.il (Y.S.); 3 Adelson School of Medicine, Ariel University, Ariel 4077601, Israel

**Keywords:** folic acid, digital interface, electronic medical records, preventive health, supplements

## Abstract

**Background:** Folic acid is essential for DNA synthesis and fetal development, with deficiency linked to anemia, cardiovascular disease and pregnancy complications. The clinical guidelines for women of reproductive age mandate supplementation as a universal preventive treatment regardless of blood folic acid levels; therefore, routine folic acid level testing is not recommended for this population. However, the vast majority of pregnant women do not implement the recommended preventive actions, indicating that new strategies are needed to improve that situation. **Objectives:** This study examined the impact of modifying the laboratory test-ordering interface in the medical record system, designed to simplify the ordering of folic acid level tests, on testing rates, deficiency detection and supplement consumption among women of reproductive age. **Methods:** This retrospective cohort analysis compared outcomes reflecting the impact of the modification on 43,952 women aged 18–42 years, assessed over one year pre- and post-integration. Statistical analyses included Chi-squared tests and logistic regression, with adjustments for age and socio-geographic status. **Results:** Post-intervention, testing rates increased from 14.74% to 17.35% (*p* < 0.0001), and deficiency detection rose from 6.30% to 7.38% (*p* < 0.0001). Supplement consumption tripled from 5.45% to 15.98% (*p* < 0.0001), with 91.37% of post-intervention consumers being new users. **Conclusions:** Modifying the presentation of tests in the laboratory test-ordering interface within electronic medical records significantly improved testing rates, enhanced deficiency detection and had a meaningful impact on treatment outcomes. These findings underscore the potential of system-level digital interventions to advance preventive care and overall health. Future research should focus on examining scalability, implementation and long-term outcomes across diverse healthcare settings.

## 1. Introduction

Electronic medical records (EMRs) have become foundational to modern healthcare systems, serving as centralized digital platforms that support the coordination, continuity, and personalization of patient care [1]. Beyond their clinical utility, EMRs facilitate a wide range of administrative and operational functions, contributing to improved reliability, safety and consistency in healthcare delivery—particularly in chronic disease management and preventive services. Integrated features such as automated alerts, reminders and evidence-based checklists help close care gaps by prompting timely interventions, including vaccinations and chronic disease screenings [1]. While studies have demonstrated favorable trends in clinical outcomes—such as improved blood pressure control, glycemic management and vaccination rates—when EMRs are effectively utilized, the evidence for direct improvements in patient outcomes remains mixed.

Despite their potential, EMRs have also introduced new challenges. Although they streamline clinical workflows by enabling real-time data access, standardized documentation and task delegation within multidisciplinary teams, they have also been associated with increased administrative burden. Physicians frequently report excessive documentation requirements, suboptimal usability and workflow misalignment, with some studies indicating that up to two-thirds of their time is spent on EMR-related tasks [2]. In response, ongoing efforts aim to enhance system usability, reduce redundant data entry and redistribute documentation responsibilities to alleviate clinician workload. In this context, the design of EMR interfaces plays a critical role in shaping physician behavior, particularly in the context of test-ordering [3].

On 15 June 2022, Leumit Health Services (LHS)—one of Israel’s four health maintenance organizations (HMOs), providing healthcare coverage to approximately 730,000 individuals—implemented a targeted modification to its EMR laboratory test-ordering interface. Prior to this change, folic acid testing could only be ordered upon a specific physician request. The update integrated folic acid testing into the standard anemia screening panel and repositioned it more prominently within the digital interface. This change was intended to reduce the administrative burden and improve accessibility for clinicians by streamlining the ordering process.

While several studies have demonstrated that increasing the complexity of test-ordering can significantly reduce utilization, sometimes by as much as 90% in the case of inappropriate test requests [4,5,6], there is comparatively limited empirical evidence on the effects of digital interface modifications that simplify and facilitate test-ordering. Understanding the impact of such system-level changes is essential for optimizing EMR design to support preventive care and evidence-based clinical decision-making.

To contextualize the significance of this intervention, it is important to consider the clinical relevance of folic acid. Folic acid, a water-soluble B-complex vitamin, is essential for DNA synthesis, amino acid metabolism and hematopoiesis. Deficiency in folic acid has been associated with macrocytic anemia, increased cardiovascular risk and a potential rise in colorectal cancer incidence. Globally, folic acid deficiency remains a significant public health concern [7]. In response, many countries have implemented mandatory food fortification policies, which have proven effective in reducing deficiency rates. For example, data from the Framingham Heart Study in the United States demonstrated a decline in folic acid deficiency prevalence from 50% to 7% following the introduction of food fortification. Similar outcomes have been reported internationally [8]. In contrast, Israel has not mandated folic acid fortification, although many food products are voluntarily enriched [9]. Historical data from a major Israeli HMO indicated that only 4.3% of individuals tested exhibited folic acid deficiency [10].

Folic acid sufficiency is particularly critical during pregnancy due to its well-established role in preventing adverse fetal outcomes, including neural tube defects such as anencephaly and spina bifida, as well as low birth weight [11,12]. While several studies have explored the association between maternal folate status and neurodevelopmental outcomes, findings remain inconclusive [13]. Notably, some evidence suggests that folic acid supplementation prior to conception may be associated with a reduced risk of autism spectrum disorder, raising important questions about the optimal timing of folate exposure and its influence on neurodevelopmental trajectories [14].

In light of these considerations, global health authorities—including the World Health Organization and the Israeli Ministry of Health—recommend a daily intake of 400 micrograms of folic acid for all women of childbearing age, regardless of laboratory test results, to ensure adequate levels during the early stages of fetal neural development [15,16,17,18].

A cross-sectional study was designed to evaluate the impact of the EMR interface modification at LHS on physician behavior regarding folic acid testing. We focused specifically on women of reproductive age, for whom there is broad consensus that routine folic acid testing is not clinically necessary [19,20]. The primary objective was to assess whether the EMR change influenced test-ordering patterns. However, the study also yielded unexpected findings related to the prevalence of folic acid deficiency and the effect of the interface change on folic acid supplement consumption. These insights may have important implications for policymakers and EMR system designers seeking to optimize preventive care delivery.

## 2. Methods

### Study Design and Sampling Strategy: A Cross-Sectional Study That Was Conducted Using Data Extracted from the Electronic Medical Records of Individuals Registered with LHS

**Research Period:** Data were collected for two distinct time periods: the year preceding the integration of folic acid testing into a standardized anemia screening panel at Leumit Health Services (15 June 2021, to 14 June 2022; defined as the pre-intervention period), and the year following the implementation (15 June 2022, to 14 June 2023; defined as the post-intervention period). The study employed an unpaired, between-subjects design. The terms “pre-intervention” and “post-intervention” were used to indicate the temporal distinction between two independent cohorts: one assessed prior to the implementation of the intervention and the other assessed following its implementation.

**Study population:** The study population included women aged 18 to 42 years who underwent folic acid level testing at LHS laboratories during the study period. This age range was selected to reflect the population most relevant to the study objectives, based on empirical data from the LHS database indicating that approximately 99% of pregnant women in the system are under the age of 42. Accordingly, this group was considered to represent the vast majority of women likely to consider folic acid supplementation as a preventive measure against fetal malformations. To ensure consistency in inclusion criteria, individuals who were older than 42 years at the end of the study period were excluded. The final cohort included 43,952 women, representing a subset of the 140,120 reproductive-age female patients registered with Leumit. Demographic and clinical characteristics of the pre-intervention group (*n* = 20,154) were compared with those of the post-intervention group (*n* = 23,798).

**Sample size calculations:** Estimates were informed by data from Vinker et al., a study on the Israeli population, which, despite a broader age range, was considered a reasonable proxy given comparable healthcare utilization patterns [10]. Based on previous studies suggesting 46% of women undergo folic acid testing, the intervention was expected to increase this rate to 51% [10]. A sample size of 1700 women tested during pre- and post-intervention periods was expected to provide at least 80% statistical power. For folate deficiency, global rates are estimated to be below 5% in developed countries, and national prevalence is approximately 4.3% [10]. Underdiagnoses in the study population were estimated to yield a 2% baseline rate. Post intervention, detection was predicted to approach 4.3%. To detect these changes, a sample size of 990 individuals in each period was estimated to achieve 80% power. For supplement consumption, U.S. studies report a 24% prevalence among women of childbearing age [20]. In this study, rates were expected to rise by 10% post-intervention due to increased testing and physician focus. A sample size of 350 individuals from both periods was projected to provide sufficient power for this analysis.

**Data analysis:** The data collected included folic acid blood levels, folic acid treatment, and demographic information. Folic acid deficiency was defined as blood levels below 3 nanograms per milliliter, in line with the National Health and Nutrition Examination Survey (NHANES) guidelines from 1988 to 1994 [21]. Folic acid treatment encompassed the purchase of prescription or over-the-counter (OTC) products containing at least 400 mcg of folic acid, purchased at least once within three months following the blood test. Demographic data included age and socioeconomic status (SES), measured on a scale of 1 to 20 as defined by the Israeli Central Bureau of Statistics based on the socioeconomic characterization of 1629 geographic units. Independent variables included folic acid blood levels, age, and SES, while the dependent variables were the rate of folic acid deficiency and the frequency of folic acid supplement consumption. Geographic location and SES were considered potential confounders and were statistically controlled using multivariate analysis, which allowed adjustment for multiple confounding variables within a single model.

**Statistical Analysis:** Statistical analyses compared the study populations before and after the intervention. Continuous demographic variables had a normal distribution and were reported as means ± SD. Outcome variables were categorical and were reported as frequencies and percentages and analyzed with Chi-squared tests and logistic regression. Adjusted analyses were conducted using a multivariable regression model, including age and socioeconomic status. The alpha level was set at 0.05 with a 95% confidence level. To better assess the intervention’s direct impact, women in the post-intervention group with prior purchases during the pre-intervention period were excluded from the analysis. This restriction ensured a more accurate evaluation of the intervention’s effect on new supplement uptake.

**Ethical Issues:** The study received ethical approval from the Helsinki Committee (Approval No. LEU-0035-23) after clearance from Leumit’s Research Committee. Data collection was conducted after all patient identifiers were removed to ensure complete anonymization prior to analysis.

## 3. Results

The study population comprised 43,952 women aged 18–42 years who underwent folic acid level testing during the research period. The baseline characteristics of the study groups before and after the intervention are summarized in Table 1.

The data indicates that there was no statistically significant difference in the mean folic acid levels between the two cohorts. However, the pre-intervention cohort was slightly older and had a higher socioeconomic status.

Table 2 presents a comparative analysis of the metrics used to evaluate the intervention: the proportion of individuals tested for folic acid blood levels, the proportion of individuals diagnosed with folic acid deficiency among reproductive-age female patients at LHS, and the proportion of individuals who purchased a folic acid-containing product, either over the counter or with a prescription, among the same population.

**Folic Acid Level Testing—**A statistically significant increase in folic acid level testing was observed following the intervention compared to the pre-intervention period, with an absolute increase of 2.61% in testing rates. This absolute increase translates to 21% more women having the test performed.

**Folic Acid Deficiency—**In the pre-intervention group, 1269 patients (6.30%) were identified with folic acid deficiency, compared to 1756 patients (7.38%) in the post-intervention group. This indicates that the post-intervention group had 19% higher odds of folic acid deficiency detection compared to the pre-intervention group. A logistic regression analysis was conducted to assess the intervention’s effect on the likelihood of deficiency detection. After adjusting for age and socioeconomic status, the multivariable regression model demonstrated that the odds of deficiency detection remained significantly elevated by 18% in the post-intervention group (OR = 1.18; 95% CI: 1.09–1.27; *p* < 0.0001).

**Folic Acid Supplement Consumption—**Prior to the intervention, 5.45% of women (*n* = 2396) purchased folic acid supplements, whereas this figure rose to 15.98% (*n* = 7022) following the intervention, indicating an absolute increase of 10.53%. Remarkably, 91.37% of post-intervention consumers (*n* = 6416) were new users, underscoring the significant impact of the intervention.

## 4. Discussion

This study evaluated the impact of modifying the digital interface at LHS to streamline the ordering process for blood folate testing. The primary objective of this intervention was to reduce the administrative burden on physicians when requesting laboratory tests. Given the well-established importance of folic acid in fetal development and the prevention of neural tube defects, the study aimed to assess whether improved accessibility to test-ordering would influence the behavior of women of reproductive age regarding adherence to folic acid supplementation guidelines.

The study results indicate a 2.61% increase in the number of women referred for folic acid testing, representing a significant 21% relative increase. It is important to note that the baseline testing rate in this age group is relatively low (14.74%, before the intervention), as treatment is typically provided regardless of blood test results. We did not find specific literature on the acceptable referral rate for folic acid testing among women of reproductive age or data for the broader population.

Among the women included in the study, the prevalence of folic acid deficiency prior to the intervention was 6.30%. Global data indicate that deficiency rates exceed 20% in many low-income countries and are typically below 5% in high-income countries [22]. However, direct comparisons are limited by the absence of standardized thresholds for defining folate deficiency and by international differences in public health policies, such as the fortification of foods and beverages with folic acid [23].

Following the intervention, there was a 19% increase in the identification of folic acid deficiency cases, suggesting that underdiagnosis may have been present prior to the digital interface modification. Although folic acid supplementation is recommended for all women of reproductive age regardless of serum folate levels, the identification of a higher-than-expected prevalence of deficiency highlights the need for enhanced efforts to promote adherence to supplementation guidelines in this population.

Before the intervention, only a small fraction (5.45%) of women adhered to the recommendations and purchased the supplement within three months following the blood test. Given that supplement purchases were tracked across most pharmacies in Israel, including over-the-counter transactions, the likelihood of missing data is minimal and the figures reasonably approximate actual consumption.

Although no studies were identified that systematically examined folic acid supplement purchases across the entire target population over a defined period, the observed supplementation rates in this study were notably lower than those reported among pregnant women [24,25]. This discrepancy may be explained by the fact that many pregnancies are planned, leading to increased awareness and proactive folic acid supplementation during the preconception period or early pregnancy, both by patients and healthcare providers [22]. However, it is important to note that the existing literature does not address the interval between the initiation of supplementation and conception, and it remains unclear how long before pregnancy folate levels influence fetal health outcomes. Given this uncertainty, and in light of the higher-than-expected prevalence of folic acid deficiency observed in this study, the gap in supplementation coverage among women of reproductive age warrants attention.

The intervention led to a modest increase of less than 5% in folic acid testing rates, while the rate of supplement purchases tripled, corresponding to an absolute increase of approximately 10%. Although a 20% rise in the detection of folate deficiency was observed, this alone is unlikely to account for the substantial increase in supplement uptake. Given that there were no significant changes in supplement availability, pricing, or national public health guidelines during the study period, these findings strongly suggest that the intervention itself was the primary driver of the observed improvements in preventive behavior.

The mechanisms underlying these outcomes merit further investigation. It is plausible that the intervention functioned in accordance with the Information–Motivation–Behavioral Skills (IMB) model [26,27,28], a theoretical framework originally developed to explain HIV-preventive behaviors and subsequently applied to a range of health-related interventions. According to the IMB model, health behavior change is influenced by the interaction of relevant information, personal motivation, and behavioral skills.

In this context, the streamlined digital interface may have encouraged physicians to initiate more frequent discussions about folic acid supplementation, thereby integrating it more seamlessly into routine care. For patients, the inclusion of folic acid testing in standard laboratory panels may have reinforced the perceived importance of the nutrient, enhancing the motivation to adhere to supplementation recommendations. Notably, this low-cost intervention achieved a 10% absolute increase in preventive health behavior without significantly increasing unnecessary laboratory testing, offering a promising strategy for improving the adherence to supplementation guidelines among women of reproductive age.

Further research is needed to examine the scalability of such interventions and to explore other potential explanations for the observed disparity between testing and supplementation outcomes. Policymakers should consider implementing similar tools to enhance the uptake of preventive health measures in diverse healthcare settings.

### 4.1. Strengths and Limitations

This study possesses several strengths that enhance its validity and applicability. It utilized a large and diverse sample, providing substantial statistical power, and evaluated a real-world digital health intervention with direct clinical relevance. The use of rigorous statistical methods further supports the robustness of the findings.

However, several limitations should be acknowledged. The study was conducted within a single healthcare organization in Israel and focused exclusively on women of reproductive age, which may limit the generalizability of the results to other healthcare systems or populations with differing public health policies or digital infrastructures. Despite statistical adjustments, socioeconomic differences between the pre- and post-intervention groups may have influenced the outcomes. Additionally, the absence of data on physician specialty limited the ability to assess provider-specific factors. The reliance on electronic medical records introduced potential biases, including the incomplete documentation of supplement adherence and unrecorded over-the-counter purchases. Furthermore, by focusing only on new supplement users, the study may have underestimated broader trends in supplementation behavior. Future research should address these gaps to better understand the mechanisms and broader impact of such digital interventions.

### 4.2. Implications and Future Directions

This study highlights the potential of digital health interventions to enhance preventive care by integrating folic acid testing into electronic medical record systems. By streamlining clinical workflows and improving accessibility, the intervention reduced barriers to diagnostic and preventive services, particularly for high-risk populations such as women of reproductive age. Although the primary aim was to simplify test-ordering, the intervention also led to the improved detection of folate deficiency and a substantial increase in supplement uptake. These findings demonstrate that even modest digital modifications can yield meaningful public health benefits.

The results suggest that similar system-level interventions could be leveraged to address other underutilized preventive measures, particularly those related to micronutrient deficiencies. Future research should examine the sustainability of increased folic acid testing and supplementation, as well as the long-term health outcomes associated with improved adherence—such as reductions in neural tube defects. Additionally, evaluating the scalability of such interventions across diverse healthcare settings is essential, with particular attention to the behavioral and systemic mechanisms that contribute to their effectiveness. These mechanisms may include changes in physician behavior, patient engagement, and institutional practices.

### 4.3. Conclusions

This study demonstrates the potential of digital health interventions to enhance preventive healthcare practices. Notably, the intervention led to significant improvements in folic acid testing rates, deficiency detection, and supplement uptake, despite current clinical guidelines not recommending routine folate testing for women of reproductive age. By addressing structural barriers through a streamlined digital interface, the intervention effectively promoted adherence to preventive supplementation, particularly among a high-risk population. These findings underscore the broader value of integrating digital tools into healthcare systems to close gaps in preventive care. Policymakers and healthcare leaders should consider the adoption of similar interventions and support further research to evaluate their scalability, sustainability, and long-term impact on health outcomes. Such efforts have the potential to advance healthcare delivery, reduce health disparities, and strengthen public health infrastructure.

## Figures and Tables

**Table 1:** Baseline characteristics of the study cohort.

Characteristic	Before	After	*p*-Value
Sample Size	20,154	23,798	
Age (mean ± SD)	29.45 ± 6.75	29.14 ± 6.89	*p* < 0.01
Socioeconomic Status	8.24 ± 3.65	7.63 ± 3.53	*p* < 0.01
Folic Acid Level (ng/mL)	7.25 ± 3.75	7.06 ± 3.81	*p* = 0.099

**Table 2:** Pre- and post-intervention outcome comparison.

Outcome	Before (*n* = 20,154)	After (*n* = 23,798)	OR [95% CI]	*p*-Value
Folic Acid Testing Rate	14.74%	17.35%	1.21 [1.19–1.24]	<0.0001
Folic Acid Deficiency (<3 ng/mL)	6.30%	7.38%	1.19 [1.10–1.28]	<0.0001
Folic Acid Supplement Use	5.45%	15.98%	3.01 [2.86–3.17]	<0.0001

## Data Availability

Datasets analyzed during the current study are not publicly available, but are available from the corresponding author upon reasonable request. The data presented in this study are available on request from the corresponding author due to privacy ethical reasons.
